# On the Cure of Hydrocele by Iodine Injections

**Published:** 1844-11

**Authors:** 


*Boston Med. and Surg. Jour.*

---

*On the Cure of Hydrocele by Iodine Injections.* By Professor Velpeau.—There are few surgical operations so easy, so simple, and which need less preparation; in what, in fact, does it consist? Of a puncture, and the injection of a certain quantity of liquid; after which the patient may be abandoned to nature, which, in general, is sufficient to effect a cure. I have operated on upwards of 300 cases of hydrocele; in my wards at the Charité I receive, on an average, from 30 to 40 patients yearly, and operate, at least, on 10 in my private practice annually; and I now declare that I never had a case in which the consecutive accidents could be attributed to the iodine injection. A patient, it is true, died, after having been operated on; but death here was caused by inflammation and purulent infiltration of the cellular tissue of the pelvis, *without communication with the tunica vaginalis.* It is, therefore, impossible to attribute this to the operation for hydrocele; nay, what is more extraordinary, especially when compared with similar cases in which the vinous injection was made, is that, on one occasion, the liquid, instead of entering the cyst, was injected in the cellular tissue, and yet did not produce sphacelus. The patients are cured by this method, on an average, in a fortnight, and I never witnessed a case of gangrene or suppuration from its use. It is therefore evident, that the iodine injection produces a degree of irritation sufficient to cure the hydrocele, but does not produce a suppurative inflammation; and this has been proved by experiments on animals, where the liquid is absorbed without causing any accidents. The same result has been obtained in cysts situated in other parts of the body; thus, about three weeks ago, I injected one on the neck, and the swelling diminished in size. It must not be supposed, however, that I assert that the iodine injection never fails; but what may be affirmed without fear of contradiction, is, that it almost always succeeds, failing only once in sixty, or even one hundred cases; unfortunately for mankind, we have but few remedies so certain in their action. This, perhaps, may be attributed to the formula I have adopted. To resume, therefore: the advantages of this method are: 1st, that the cure is almost always obtained; 2d, that the gangrene does not take place, even when the injection enters the cellular tissue; 3d, that the operation is sooner done than that in which the vinous injection is employed, since it is not necessary to heat the liquid, or to leave it a certain time in the cyst, &c. If, after the operation, you wish to hasten the resolution, the patient must be treated as for orchitis, and since scarifications with a lancet are useful in the latter, they will equally be so in the phlegmasia which follows the injection. In the generality of cases, this, however, is not requisite, the patient recovering without any remedy being employed; it, perhaps, takes eight or ten days before he is quite well, but this is not of much importance, as his sufferings are slight. Finally, I have remarked that the iodine injection is a powerful discutient when hydrocele is complicated with *engorgement* of the testicle or of the epididymis.—*London Med. Times.*

The formula employed by Professor Velpeau is the following: ℞. Tinct. iodin. ℥j. Aque distillatæ, ℥ij. M.—*Boston Med. and Surg. Jour.*, July 22, 1844.

